# Rheumatoid Arthritis from Easy to Complex Disease: From the “2022 GISEA International Symposium”

**DOI:** 10.3390/jcm12082781

**Published:** 2023-04-09

**Authors:** Simone Perniola, Maria Sole Chimenti, Francesca Romana Spinelli, Bruno Frediani, Rosario Foti, Sara Ferrigno, Cristina Garufi, Giulia Cassone, Vincenzo Venerito, Fabiola Atzeni, Roberto Caporali, Fabrizio Conti, Ennio Giulio Favalli, Florenzo Iannone, Marco Sebastiani, Gian Franco Ferraccioli, Giovanni Lapadula, Elisa Gremese

**Affiliations:** 1 Immunology Research Core Facility, Gemelli Science and Technology Park (GSTeP), Fondazione Policlinico Universitario A. Gemelli IRCCS—Rome, 00168 Rome, Italy; 2 Division of Clinical Immunology, Fondazione Policlinico Universitario A. Gemelli IRCCS, Università Cattolica del Sacro Cuore—Rome, 00168 Rome, Italy; 3 Rheumatology, Allergology and Clinical Immunology, Department of “Medicina dei Sistemi”, University of Rome Tor Vergata, 00133 Rome, Italy; 4 Dipartimento di Scienze Cliniche Internistiche, Anestesiologiche e Cardiovascolari-Reumatologia, Sapienza Università di Roma, 00185 Rome, Italy; 5 Department of Medical Sciences, Surgery and Neurosciences, University of Siena, 53100 Siena, Italy; 6 Rheumatology Unit, San Marco Hospital, Policlinico University of Catania, 95124 Catania, Italy; 7 Rheumatology Unit, Azienda Ospedaliera Policlinico di Modena, University of Modena and Reggio Emilia, 41121 Modena, Italy; 8 Department of Emergency and Organ Transplantation, Rheumatology Unit, University of Bari, 70121 Bari, Italy; 9 Rheumatology Unit, Department of Experimental and Internal Medicine, University of Messina, 98122 Messina, Italy; 10 Department of Rheumatology and Clinical Sciences, ASST Gaetano Pini-CTO, 20126 Milan, Italy; 11 Department of Clinical Sciences and Community Health, University of Milan, 20122 Milan, Italy

**Keywords:** rheumatoid arthritis, seronegative, seropositive, remission, therapy

## Abstract

Rheumatoid Arthritis (RA) is a systemic disease with many different clinical phenotypes. RA could be classified according to disease duration, seropositivity for rheumatoid factor (RF) and/or anti-citrullinated protein antibodies (ACPA), joint subtype, clinical behaviourbehavior and many other subgroups. In this review, we summarize and discuss the multifaceted aspects of RA, focusing on the relationship between autoimmunity status and clinical outcome, achievement of remission and influence on treatment response, from the 2022 International GISEA/OEG Symposium.

## 1. Introduction

The “Italian Group for the Study of Early Arthritis” (GISEA) includes 21 hospital and community-based Rheumatology Units throughout Italy. It has developed and maintained a nationwide registry to promote the study of patients with inflammatory arthritis according to standard-of-care criteria [1]. The annual International GISEA Meeting aims to explore the state of art in many fields of rheumatology, in particular, rheumatoid arthritis (RA), psoriatic arthritis (PsA), and axial spondylarthritis (SpA) and joint involvement in connective tissue diseases. The present review derives from the 2022 meeting session titled “Rheumatoid Arthritis, from easy to complex disease”. The session aimed to summarize and discuss the multifaceted aspects of RA, focusing on the relationship between autoimmunity status and clinical outcome, treatment response and achievement of remission.

RA is a systemic disease, including different clinical phenotypes. As for many rheumatic diseases, RA could be defined as a spectrum of disease with many different facets. 

Early RA is defined by the ACR guidelines as a disease duration of less than 6 months from the onset of symptoms [2]. However, the time frame to distinguish early from established RA varies among different studies from a few weeks to 1–8 years. On the other hand, no consensus has been reached by the scientific community to define established RA. There is also debate about definition of early or established RA, as many different modalities have been proposed, for example chronological or radiographical definition, classification based on clinical outcome (e.g., remission), or based on disease development [3].

An important subgroup of established RA patients is the refractory or “difficult- to-treat (D2T)” disease, which was defined by EULAR in 2020 [4].

Over the past 20 years RA has moved from being an inexorably disabling and quality-of-life limiting disease to a manageable long-term condition. The key to this improvement has been the early and aggressive treatment of inflammation and the increasing number of available both conventional synthetic (cs-) and biological (b-) and targeted synthetic (ts-) disease modifying antirheumatic drugs (DMARDs) for patients refractory to the therapy [5,6,7]. 

RA could also be classified according to seropositivity for rheumatoid factor (RF) and/or anti-citrullinated protein antibodies (ACPA), arising controversy regarding the presence of different clinical course and response to therapy between the seropositive or seronegative disease. Moreover, RA can manifest itself as a small joint subtype, total joint subtype, systemic subtype and overlapping subtype.

Finally, comorbidities play a very important role in the clinical course of the disease, primarily in terms of prognosis and response to therapy.

## 2. Is Seronegative RA Easy or Complex to Treat?

Seronegative RA is defined by the absence of RF and ACPA. The prevalence of seronegative RA varies according to the study population, and divergence may be attributed to differences in the patients’ selection, but mainly up to 20–30% of patients recruited into RA cohorts and clinical trials have seronegative RA [8,9]. Clinical presentation, treatment response rates and disease course of seronegative RA are usually considered less severe than for seropositive RA; however, literature data on this topic are conflicting. 

Autoantibodies, mainly ACPA, are thought to play a key role in RA pathogenesis; nonetheless, it seems evident that the knowledge about the pathogenesis, treatment response and clinical course of seronegative RA, is still limited [10,11]. 

In this section, we discuss the common belief that seronegative RA is easier to manage than seropositive RA, analyzing the scientific evidence that supports or disproves this widespread opinion.

In the recommendations for RA management, seropositivity is included among the poor prognostic factors, together with a persistently active disease, failure of multiple cs-DMARDs and presence of early bone erosions [7]. In the derivation study that led to the drafting of the 2010 ACR/EULAR classification criteria for RA, moderate or high ACPA titer was the main predictive factor for the initiation of a cs-DMARD [12]. 

Although 2010 ACR/EULAR classification criteria were aimed to improve the classification of arthritis as RA at its early stages, their sensitivity in seronegative RA patients was low [13,14]. It could account for some variability in identification of seronegative RA cases in population-based studies using different criteria sets [15]. Myasoedova and colleagues emphasized differences in RA occurrence and prevalence in the last years with associated differences in clinical patterns and seropositivity. In particular, they suggested a different trend in the incidence of RA by serologic status, in a population-based inception cohort of individuals aged ≥18 years who first fulfilled the ACR 1987 criteria for RA between 1985 and 2014. The hypothesis was that the decreased incidence of RF-positive and the rise in RF-negative RA resulted from a change in environmental factors; the decline in RF-positive RA may have coincided with a significant decrease in the prevalence of current or former smoking and an increase in rate of never smokers [15]. Moreover, the increased prevalence of obesity could have contributed to the observed increase in RF-negative RA.

Some authors suggested a decrease in the incidence of RF-positive RA in 2005–2014 as compared to the previous decades. A decline in the incidence of RF-positive RA had been already reported in Finland in 1980–2000 [16], and previously in the Pima Indian population, also in younger birth cohorts [17]. These findings may reflect a potential decline in RA severity in association with advance in RA treatment over time. However, no changes in the incidence or prevalence of RA by serologic status have been reported thus far in the US population, including the population of Olmsted County, MN, where the proportion of RF-positive and RF-negative RA cases remained largely unchanged since 1955 [8,15].

EULAR did not include serological status among classification criteria of difficult-to-treat (D2T) RA [4]. Hence, the threshold for earlier and more intensive disease-modifying treatment may be lower in seropositive patients. For similar disease activity, a precocious and aggressive therapy could account for a greater long-term improvement in disease activity and less functional disability reported in seropositive RA of the Leiden cohort [18]. 

In an inception cohort of 234 patients—of whom 15.3% seronegative—evaluated in the first 2 years from symptoms onset, patients with seronegative RA tend to have a higher disease activity as assessed by clinical evaluation and ultrasounds; however, radiographic damage and patient-reported outcomes were similar between the seropositive and seronegative groups [10]. Moreover, treatment target seems to be achieved more often by seronegative RA patients. By 5 years, 50% of seropositive compared with 28% of seronegative patients achieved the remission at least once; this corresponds to a nearly 3-fold difference in achieving remission at least once (hazard ratio: 2.91; 95% CI 1.34–6.31) for seropositive versus seronegative patients [10].

Most of the literature data suggest that ACPA positivity is associated with a worse clinical outcome. Despite no difference in demographic data, and a similar number of tender and swollen joints, seronegative RA of the ESPOIR early arthritis cohort had lower acute phase reactants value, lower disease activity, less disability and less erosions. Even after 3 years of follow-up, patients with seronegative RA patients had significantly less active disease and radiographic progression despite significantly less frequent use of b-DMARDs [19]. ACPA negativity seems also to reduce the risk of drug discontinuation. Among 1237 patients of the Japanese registry, those who were seronegative had significantly lower discontinuation rate due to insufficient response to therapy [20]. Furthermore, seronegative patients had a significantly lower disease activity at baseline and a greater chance to achieve remission at 12 months (46.0% of RF-negative and 36.7% of RF-positive patients; *p* < 0.05; 45.5% of ACPA-negative and 39.2% of ACPA positive patients, *p* = ns) [20].

Thus, serological status seems to affect differently the target achievement. Among 535 patients with early RA described by Bugatti et al., ~25% achieved a near-remission status at 6 and 12 months. While ACPA negativity was and independent predictor of Patients Global Assessment near-remission, seropositivity was independently associated with near-remission for swollen joint count >1 [21]. 

Another clue in favor of the hypothesis that seronegative RA is a less complex disease to treat comes from the RETRO study (Reduction of Therapy in patients with Rheumatoid arthritis in Ongoing remission). Evaluating different strategies of csDMARDs or bDMARDs discontinuation in RA patients with persistent remission, the Authors found that ACPA were associated with disease relapse after treatment discontinuation [22]. 

The previously mentioned epidemiological study showing a decline in RF-positive RA also showed that radiographic progression within the first year after the diagnosis of RA were more common in the decade 2005–2014, when the RF positive disease was more common than the seronegative one. X-ray detectable erosions were as common in RF-negative as in RF-positive RA patients [15]. Despite the higher disease activity reported by Fedele et al. in seronegative RA, ACPA was still the strongest risk factor for the development of radiographic progression among 408 patients with early RA [23]. However, seronegative RA could be an erosive, potentially disabling disease such as the ACPA-positive subtype.

Comorbidities and extra-articular manifestations are recognized as main contributors to D2T RA [24]. Among them, interstitial lung disease and accelerated atherosclerosis seems to be associated with ACPA positivity [25,26,27]. This aspect can make the management of seropositive RA somewhat more challenging. However, in a recent study evaluating the phenotypical difference between seronegative and seropositive RA patients, the authors did not detect any differences regarding cardiovascular risk in a 10-year follow-up [28]. On the other hand, a recent metanalysis showed a higher risk to develop interstitial lung disease for seropositive RA patients [29]. 

The lack of association between improvement of disease activity and long-term outcomes detected in the cohort described by Matthijsenn et al. may reflects a different underlying pathogenesis of seronegative compared to the seropositive RA. Although the differences in the pathogenetic mechanisms of seronegative and seropositive RA are not yet fully understood, different genetic background and environmental factors seems to converge in a different immunopathogenesis as discussed by De Stefano et al.. in their recent literature review [30].

Seronegative arthritis includes a heterogeneous group of various disease entities and can pose diagnostic challenges. However, it should not be ignored that seronegative arthritis is not always actually RA. Among 9784 patients diagnosed with seronegative RA, 564 had a subsequent new diagnosis of Spondylarthritis including PsA, non-radiographic-axial SpA, ankylosing spondylitis, or inflammatory bowel disease-associated SpA; overall, the cumulative prevalence of SpA diagnosis in this population was 8.8% during a 15-years follow up. Male gender and younger age at the time of diagnosis were associated with greater probability to reclassification from seronegative RA to SpA [9]. The challenge of the differential diagnosis could account for the diagnostic delay described in seronegative RA patients. Diagnostic and treatment delay due to referral lag, wrong diagnosis and improper treatment have also been described. Patients with seronegative RA have a significant later specialist referral and the time from first joint swelling to the clinical diagnosis of RA was significantly longer in seronegative RA patients [11].

Finally, elderly onset RA, especially its polymyalgia-like phenotype with proximal joints involvement, is often seronegative making the differential diagnosis with Polymyalgia Rheumatica challenging; as a consequence, older patients with seronegative RA may in-cur a delay in diagnosis and access to the correct therapies [31].

The use of synovial biopsy or radiodiagnostic tools such as ultrasound (US) of the inflamed joints may better elucidate different clinical phenotypes of the RA or may help to differentiate seronegative RA from other inflammatory arthropathies. Ultrasound is a noninvasive, cost-effective method that can visualize pathophysiological changes such as synovitis, tenosynovitis, enthesitis, bone erosions, and crystal deposits at a subclinical level, which makes it an effective technique to identify and differentiate most common types of inflammatory arthritis [32]. For example, US evaluation suggest that seropositive patients may have more synovitis and vascularization, especially in the metacarpal-phalangeal joints [28,33].

In conclusion, the easiness or complexity of treating seronegative RA should be relativized and compared with the seropositive counterpart. While ACPA positivity is an unfavorable prognostic factor, late referral and diagnostic delay due to challenging differential diagnosis may affect the long-term outcome of RA patients. Seronegative RA should be considered as a heterogeneous clinical entity that needs the same timely referral to rheumatologist as the seropositive RA, to improve the long-term prognosis of seronegative RA patients. Indeed, diagnostic and therapeutic delay are the major risk factors for worse clinical and radiographic outcomes. 

## 3. How Achievable Is Remission in Current RA Management and Is It Easy to Maintain?

Remission is the main goal of the RA therapy and it remains the real target of the RA management, preventing joint damage and disability [34]. To date, the broader pharmacological armamentarium significantly increased rheumatologist’s chances of controlling the disease and achieving clinical remission in RA. However, there is not only one univocal definition of remission and it remains a very heterogeneous state, which would require an effort for a clear and precise definition [35]. In particular, Boolean remission stratifies RA patients into a deeper grade of remission than Simple Disease Activity Index (SDAI) or Clinical Disease Activity Index (CDAI) remission definition, which in turn give a more stringent grade of remission than Disease Activity Score (DAS) or DAS28 [36]. Moreover, recently Boolean remission definition was revised by ACR/EULAR increasing the PtGA threshold from 1 (Boolean1.0) to 2 (Boolean2.0) (within a range of 0–10) to improve the agreement with index-based remission criteria without compromising the predictive value for radiographic or functional outcomes [37] 

The different grade of remission gives the opportunity to sort RA patients and to adequately treat them, reducing the risk of joint damage and damage progression [38]. Due to the high grade of heterogeneity in clinical remission definitions, disease remission in RA is also defined by other non-clinical parameters. In particular, by mean of US examination, it is possible to define subclinical synovitis as a condition satisfied by clinical criteria of remission but in which synovitis is present as hypertrophy of the synovial membrane and Power Doppler signal, influencing clinical outcomes, disease flares and therapeutic strategy [39,40]. 

The definition of subclinical synovitis has therefore opened a breach in the concept of remission, pushing the rheumatologists beyond just clinical evaluation to find a new and deeper concept of remission. In this scenario, synovial tissue (ST) evaluation has acquired increasing significance [41]. Recently, Alivernini et al. showed that a simple ST histological analysis, evaluated by the Krenn synovitis score (KSS), could add important information to the management of RA [42]. KSS is a feasible hematoxylin and eosin-based staining system which enables discrimination between low- and high-grade synovitis [43]. In 240 naïve to treatment RA patients, baseline KSS, evaluated in ST samples obtained from minimally invasive US-guided biopsies of large joints, is synergistically associated with disease duration and activity in predicting the DAS-remission achievement at 6 months follow-up [40]. Therefore, the histological ST assessment can be a first tool to help the identification of important prognostic factors of the disease.

Due to RA heterogeneity and b-DMARD distinct modes of actions, it will be increasingly necessary to find and adopt other specific biomarkers which might support clinical decisions and predict treatment response. An example is the recent prospective longitudinal study conducted by Gremese et al., investigating the impact of CTLA4-Ig treatment on peripheral blood-derived CD4^pos^ cells in RA patients. The authors found that CTLA4-Ig modulates peripheral blood-derived CD4^pos^ cell subtypes in RA patients in terms of Th17 CD4^pos^ cells decrease, a trend toward the normalization of Tregs and an effect on IL-6 burden. Moreover, pre-treatment IL-6 serum levels and CD4^pos^CD25^pos^FoxP3^pos^ rates arose as putative biomarkers of successful treatment response to CTLA4-Ig in RA patients [44]. In addition to the unmet need of identifying validated biomarkers of treatment response, to date another important aspect is the search for biomarkers able to define the achievement and maintenance of remission in RA. Despite the seemingly simple concept of remission, the biological mechanism of dampening inflammation is regulated by a complex interplay of many molecules and mechanisms. Among them, Specialized Pro-resolving Mediators (SPMs) seem very important in the inflammation resolution. SPMs are bioactive metabolites derived from omega-3 essential fatty acids such as Eicosapentaenoic Acid (EPA) and Docosahexaenoic Acid (DHA), among which Resolvin D- and E-series, Protectins, Maresins and Lipoxins (belonging to Arachidonic Acid (AA) metabolites) have been identified so far [45,46]. SPM counter-regulate pro-inflammatory chemical mediators, reducing magnitude and duration of inflammation, and stimulate reepithelialisation, wound healing, and tissue regeneration in model organisms [46]. SPM act on multiple immune and stromal cells by binding to specific surface receptors as E-Resolvin Receptor 1 (ERV1, also known as ChemR23 or CMKLR1) and Leukotriene B4 Receptor 1 (BLT1, also known as LTB4R) for Resolvin E1, Formyl Peptide Receptor 2 (ALX/FPR2) for Resolvin D1 and Lipoxin A4 and G Protein-coupled Receptor 32 (GPR32 also known as DRV1) for Resolvin D1. 

Although there are no clear data on plasma lipid metabolites levels in RA patients [47,48], the importance of DHA and EPA metabolites in the pathophysiology of RA at disease onset has been demonstrated [49,50]. Furthermore, a down-regulation of ALX/FPR2 and BLT1 receptors in peripheral blood CD14^pos^ cells relating to RA disease activity suggests that SPM and their receptors seem to have a role also in the remission phase in RA and could be putative biomarkers of inflammation resolution in this disease [51].

**Figure 1:**
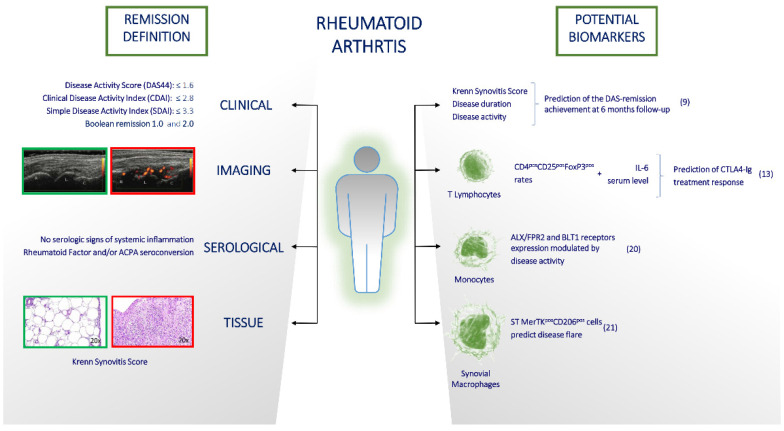
How remission status in Rheumatoid Arthritis is defined (left section) and what are the next potential biomarkers (right section) which might help in the management of this disease.

Finally, maintaining the remission status in RA means avoiding disease flares. In this scenario, finding a biomarker that predicts loss of remission could help the RA therapeutic management. To date, a promising study identified KSS and synovial tissue macrophages (STMs) phenotype as potential biomarkers of an increased risk of flare after treatment de-escalation in patients with RA in remission. In particular, digital spatial profiling analysis, using CD68 macrophage morphology markers, revealed that ST lining and sub-lining layer CD68^pos^ spatial transcriptomics distinguished, at baseline, remission RA who flared after treatment modification from those who did not (Figure 1) [52]. 

To date Rheumatoid Arthritis (RA) remission can be defined in many ways: by clinical (using DAS28, CDAI/SDAI and Boolean indexes), ultrasound (presence/absence of synovial membrane hypertrophy and Power Doppler signal), serological and tissue (using Krenn Synovitis Score, KSS) examination. In this scenario, baseline KSS predicts the DAS-remission achievement at 6 months follow-up [39]. Due to RA heterogeneity and b-DMARD distinct mechanisms of actions, it will be increasingly necessary to find specific biomarkers which might support clinical decisions, predicting treatment response. An example is pre-treatment IL-6 serum levels and CD4^pos^CD25^pos^FoxP3^pos^ rates arose as putative biomarkers of successful treatment response to CTLA4-Ig in RA patients [43]. Furthermore, a down-regulation of Specialized Proresolving Mediators (SPM) ALX/FPR2 and BLT1 receptors in peripheral blood CD14^pos^ cells suggest a putative role of those receptors as biomarkers of inflammation resolution in RA [50]. Finally, maintaining the remission status in RA means avoiding disease flares. In this scenario, digital spatial profiling analysis, using MERTKposCD206pos macrophage morphology markers, revealed that MERTKposCD206pos spatial transcriptomics distinguished, at baseline, remission RA who flared after treatment modification from those who did not [51].

In conclusion, disease remission in RA is defined by multiple levels and each remission level has peculiar pathogenetic aspects with different potential biomarkers, which could be related to specific therapy or disease phase. The best chance of achieving and maintaining the remission status in RA is to reach a deep biological remission.
